# Injectable PEG-PCL-PEG Copolymers for Skin Rejuvenation: In Vitro Cell Studies to in Vivo Collagen Induction

**DOI:** 10.3390/polym17141892

**Published:** 2025-07-08

**Authors:** Seunghwa Lee, Aram Kim, Jimo Koo, Yunsik Kim, Sunglim Choi, Jin Cheol Cho

**Affiliations:** 1 R&D Center, CHA Meditech. Co., Ltd., 119 Techno 2-ro, Yuseong-gu, Daejeon 34116, Republic of Korea; lshlsh0106@chamc.co.kr (S.L.); slchoi85@chamc.co.kr (S.C.); 2 Department of Biomedical Science, School of Medicine, CHA University, Seongnam 13488, Republic of Korea; tmfdkdjssl@hanmail.net; 3 Research & Development Center, CHA Advanced Research Institute, Seongnam 13488, Republic of Korea; koochj@chamc.co.kr; 4 Consumer Health 2 Center, CHA Advanced Research Institute, CHA Bundang Medical Center, CHA University School of Medicine, Seongnam 13496, Republic of Korea; spcp@chamc.co.kr

**Keywords:** PEG-PCL-PEG copolymers, solubilization, skin rejuvenation, idebenone, antioxidant activity, collagen regeneration

## Abstract

In this study, we designed an injectable skin-rejuvenating formulation based on polyethylene glycol–polycaprolactone–polyethylene glycol (PEG-PCL-PEG) copolymers to provide a synergistic combination of biocompatibility, antioxidative capacity, and regenerative potential. Through the systematic optimization of the precursor molar ratio and molecular weight, well-defined PEG-PCL-PEG copolymers were synthesized and structurally characterized using gel permeation chromatography (GPC), proton nuclear magnetic resonance (^1^H-NMR), and Fourier transform infrared (FT-IR) spectroscopy. An optimized precipitation and drying protocol effectively reduced residual solvents, as confirmed by gas chromatography (GC). Idebenone was incorporated as an antioxidant to prevent skin aging, while hyaluronic acid (HA), L-arginine, and glycerin were included to promote collagen regeneration. In vitro assays demonstrated that idebenone-loaded samples exhibited prolonged intracellular antioxidant activity with low cytotoxicity. The collagen-promoting formulation, containing HA, glycerin, and L-arginine, enhanced the expression of transforming growth factor-β (TGF-β) and type III collagen (COL3) while suppressing inflammatory genes, suggesting a favorable environment for extracellular matrix remodeling. In vivo evaluation corroborated these outcomes, showing angiogenesis, collagen reorganization, and progressive dermal thickness. Histological analysis further confirmed sustained matrix regeneration and tissue integration. These results highlight the potential of PEG-PCL-PEG-based injectables as a multifunctional platform for collagen regeneration, offering a promising strategy for both cosmetic and clinical applications.

## 1. Introduction

As global demand for non-invasive anti-aging therapies continues to grow, the development of advanced biomaterials that support comprehensive skin regeneration has garnered significant attention [1]. The global market for non-invasive esthetic procedures, including injectable and topical anti-aging treatments, was valued at USD 60.8 billion in 2022 and is projected to reach USD 110.6 billion by 2030, reflecting a compound annual growth rate of 7.9% [2]. Among various biodegradable polymers, polycaprolactone (PCL) has been widely used due to its favorable biocompatibility and slow degradation rate, yielding low-toxicity byproducts [3,4]. However, its intrinsic hydrophobicity limits its utility in aqueous environments and topical formulations. To address this limitation, amphiphilic copolymers incorporating hydrophilic components such as PEG have been developed [5,6,7]. These hybrid systems improve water dispersibility, enhance cellular compatibility, and provide chemical versatility for functional modification [8,9,10,11].

In the field of skin health and cosmetic dermatology, several bioactive agents have emerged as effective adjuncts to polymeric delivery platforms. HA, a naturally occurring glycosaminoglycan in the extracellular matrix, plays a critical role in maintaining skin hydration and elasticity due to its remarkable water-binding capacity [12,13,14]. It also strengthens the skin’s barrier and reduces transepidermal water loss, making it a key component in dermatological formulations [15,16].

L-arginine, a semi-essential amino acid, involved in multiple metabolic pathways, facilitates tissue repair by promoting collagen synthesis and stimulating the expression of growth factors. Its vasodilatory effects further enhance local circulation, creating a microenvironment favorable for cellular regeneration [17,18,19].

Idebenone, a synthetic analog of coenzyme Q10, exhibits potent antioxidant activity by scavenging reactive oxygen species (ROS) and protecting the extracellular matrix from oxidative stress [20,21]. Additionally, idebenone inhibits tyrosinase-mediated melanin synthesis, contributing to skin-brightening effects and offering dual cosmetic benefits.

To effectively harness the therapeutic potential of HA, L-arginine, and idebenone, an appropriate delivery platform is essential to ensure sustained release, bioactivity preservation, and spatial retention in target tissues [22]. Conventional carriers such as PLGA and HA-based systems have been widely explored for these purposes; however, their application is often limited by issues such as acidic degradation products in PLGA or rapid enzymatic breakdown and mechanical weakness in HA formulations [23,24]. To address these limitations, recent developments in polyurethane-urea-based biodegradable scaffolds have demonstrated that tuning polymer backbone chemistry—particularly through the incorporation of urethane bonds—can significantly enhance the mechanical resilience and bioactivity of regenerative systems in vivo. These findings underscore the growing interest in urethane-linked soft biomaterials as adaptable platforms for tissue integration and controlled therapeutic delivery [25,26]. Building on these advances, amphiphilic copolymer systems such as PEG-PCL-PEG tri-block copolymers have attracted attention for their tunable hydrophilic–hydrophobic balance, sustained in vivo stability, and reduced inflammatory response [27,28]. These characteristics make PEG-PCL-PEG systems well-suited to support the regenerative and antioxidant activity of incorporated agents, particularly in minimally invasive applications requiring both biocompatibility and structural resilience [29].

Based on these insights, this study presents the development and evaluation of an injectable PEG-PCL-PEG-based polymeric system incorporating HA, L-arginine, and idebenone to simultaneously address oxidative stress and promote dermal regeneration. PEG-PCL-PEG copolymers were synthesized and structurally characterized to confirm their compositional integrity. Functional formulations were prepared by selectively incorporating antioxidant or regenerative agents, and their biological efficacy was assessed through a combination of in vitro and in vivo assays.

Specifically, HA- and L-arginine-containing formulations were evaluated for regenerative activity using histological and gene expression analyses in a hairless mouse model. Idebenone-loaded formulations were assessed for antioxidant performance via oxygen radical absorbance capacity (ORAC) assays. By isolating and systematically evaluating the contributions of each functional component, this study establishes a modular platform for the development of customizable skin therapeutics. These findings underscore the therapeutic potential of multifunctional injectable systems and lay the foundation for future applications in cosmetic and clinical dermatology requiring targeted biological activity.

## 2. Materials and Methods

### 2.1. Materials

Polyethylene glycol monomethyl ether 2000 (PEG, M_n_ ≈ 2000) was purchased from Tokyo Chemical Industry Co., Ltd. (Tokyo, Japan). Idebenone, 2,2’-azobis(2-methylpropionamidine) dihydrochloride (AAPH), ε-caprolactone (CL), hydrogen chloride solution in diethyl ether (1.0 M HCl·Et_2_O), hexamethylene diisocyanate (HMDI), pyrene, and phosphate-buffered saline (PBS) were obtained from Sigma-Aldrich (St. Louis, MO, USA). Organic solvents, including toluene and dichloromethane, were sourced from Samchun Pure Chemical Co., Ltd. (Pyeongtaek, Republic of Korea). Fetal bovine serum (FBS) was acquired from Thermo Fisher Scientific, Inc. (Waltham, MA, USA). Primary human dermal fibroblast (HDF) cells were obtained from Lonza (Basel, Switzerland). HA powder was supplied by Inno Biotechnology Co., Ltd. (Qufu, China). Glycerin was purchased from Acidchem International Sdn. Bhd. (Penang, Malaysia), and L-arginine was acquired from Ajinomoto Co., Inc. (Saga, Japan).

### 2.2. Synthesis of PEG-PCL-PEG Copolymers

To synthesize the PEG-PCL copolymer, 500 g of PEG was dissolved in 2.5 L of toluene in a 15 L glass reactor (Buchi, Uster, Switzerland). The mixture was gradually heated to 60 °C while stirring continuously at 125 rpm. After complete dissolution, the solution was subjected to the same conditions. Subsequently, 1 kg of CL was added, and stirring was continued to ensure homogeneity. The molar feed ratio of PEG to caprolactone was set at 1:35.7, which was determined to be optimal for achieving amphiphilic balance and colloidal stability in the final copolymer formulation. Following this step, 485 mL of hydrogen chloride solution in diethyl ether (HCl·Et_2_O) was added to initiate polymerization. The reaction was maintained for 24 h at 60 °C with constant stirring. After the completion of the reaction, 1 kg of dichloromethane was added to facilitate the uniform dispersion of the resulting copolymers. The solution was then precipitated by dropwise addition into cold diethyl ether (0 °C) under continuous stirring. The precipitated copolymers were collected by filtration and thoroughly washed with cold ether to remove unreacted monomers and residual solvents.

**Figure 1:**
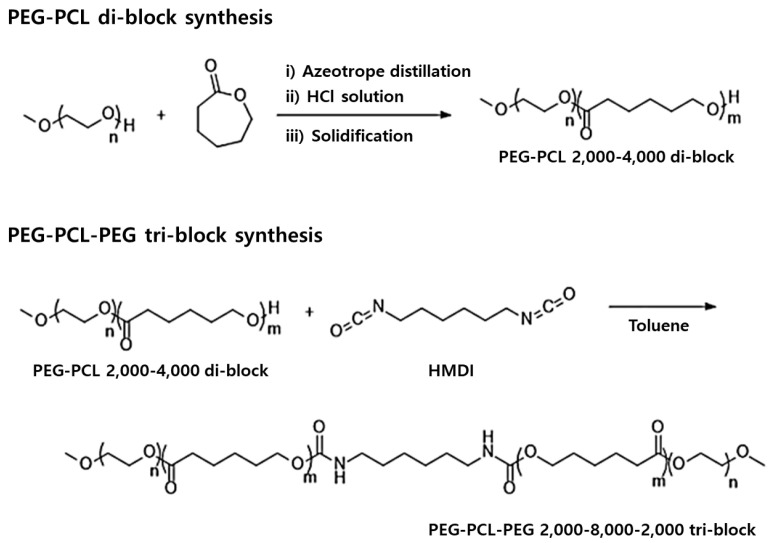
Schematic representation of urethane coupling reaction between PEG-PCL di-block copolymers and HMDI, yielding PEG-PCL-PEG tri-block copolymers via urethane linkage formation.

For the synthesis of the PEG-PCL-PEG tri-block copolymers, 900 g of the purified PEG-PCL copolymer was redissolved in 3 L of toluene in the same reactor. Then, 12 g of hexamethylene diisocyanate (HMDI) was subsequently added, and the reaction was stirred at 180 rpm for 24 h. To minimize undesirable side reactions such as the crosslinking or hydrolysis of isocyanate groups, the reaction was conducted in toluene under a dry nitrogen atmosphere, which helped to limit moisture interference during urethane bond formation. After completion, the mixture was condensed, and 1.5 L of dichloromethane was added while maintaining stirring. The solution was then precipitated dropwise into cold ether (0 °C) under agitation. The final product was isolated by filtration and dried under vacuum for 48 h to yield the PEG-PCL-PEG copolymers. The reaction mechanism is illustrated in Figure 1, where two equivalents of PEG-PCL-OH react with HMDI to yield the symmetric PEG-PCL-PEG copolymer. The tri-block copolymer was synthesized by urethane linkage formation, wherein the terminal hydroxyl groups of PEG-PCL react with the electrophilic isocyanate groups of HMDI to form covalent -NH-COO- bonds. This step-growth reaction enables the formation of the symmetric PEG-PCL-PEG copolymer.

### 2.3. Preparation of Functional Agent-Loaded Formulations

In addition to the pilot-scale synthesis of the P5 sample described above, formulations P1–P4 were prepared at laboratory scale using the same general procedure, with variations in the composition of functional additives to assess their individual or combined effects. Formulations P2–P4 incorporated idebenone at increasing concentrations, and P4 additionally contained sodium hyaluronate (HA), while P5 included HA, L-arginine, and glycerin to enhance collagen-regenerative potential. All formulations were prepared by sequentially dispersing each component into 100 mL of DI water under magnetic stirring at room temperature. The PEG-PCL-PEG copolymer was first dissolved until the solution became visually clear, followed by the addition of idebenone, sodium hyaluronate, glycerin, and L-arginine, depending on the formulation. Each component was added according to the predetermined % (*w*/*v*) values. For instance, formulation P3 consisted of 10.0 g PEG-PCL-PEG, 0.1 g idebenone, 0.1 g sodium hyaluronate, 1.0 g glycerin, and 0.1 g L-arginine, all dissolved in 100 mL of DI water to yield a homogeneous injectable solution. The same procedure was applied across P1–P4 by varying only the additive composition.

### 2.4. Physicochemical Characterization

#### 2.4.1. Gel Permeation Chromatography

The average molecular weights (M_w_) of the PEG-PCL and PEG-PCL-PEG copolymers were determined using a GPC system (ACQUITY APC, Waters, Milford, MA, USA) equipped with a series of four Styragel columns (HR-0.5, HR-1, HR-4E, and HR-4). Samples were prepared by dissolving the copolymers in tetrahydrofuran (THF) at a concentration of 2 mg/mL, followed by filtration through a 0.45 µm membrane filter to remove particulates. Chromatographic separation was performed at a flow rate of 1.0 mL/min. Polystyrene standards were used to calibrate the system for molecular weight estimation.

#### 2.4.2. ^1^H-Nuclear Magnetic Resonance and Fourier Transform Infrared Spectroscopy

The chemical compositions of the PEG-PCL and PEG-PCL-PEG copolymers were confirmed by ^1^H-NMR spectroscopy using a Fourier 300 spectrometer (Bruker, Billerica, MA, USA). Samples were dissolved in deuterated chloroform (CDCl_3_) and analyzed at 24 °C. FT-IR spectroscopy was additionally performed to investigate the chemical structures and to identify characteristic functional groups in the copolymers. FT-IR spectra were recorded using a Spectrum 2 instrument (PerkinElmer, Waltham, MA, USA), with data collected over 16 scans at a resolution of 8 cm^−1^.

#### 2.4.3. Differential Scanning Calorimeter

The thermal behaviors of the PEG-PCL and PEG-PCL-PEG copolymers were analyzed by DSC using a DSC1 instrument (Mettler-Toledo, Greifensee, Switzerland). Samples were heated at a rate of 10 °C/min under a nitrogen atmosphere to prevent oxidative degradation during measurement.

#### 2.4.4. Gas Chromatography

A residual solvent analysis of the final PEG-PCL-PEG copolymer was conducted by GC in accordance with ICH Q3C guidelines. Diethyl ether, dichloromethane, and toluene, solvents used during the synthesis and purification processes, were quantified using a GC system equipped with a flame ionization detector (FID) and a DB-624 capillary column (30 m × 0.32 mm × 1.8 µm). Approximately 100 mg of the polymer sample was dispersed in N,N-dimethylformamide (DMF), sealed in a headspace vial, and incubated at 80 °C for 45 min. GC analysis was performed with an oven temperature starting at 40 °C (held for 5 min), followed by an increase to 240 °C at a rate of 10 °C/min. Helium was used as the carrier gas at a flow rate of 1.5 mL/min. Solvents were identified and quantified by comparison with certified reference standards, and the method met the sensitivity requirements specified by the ICH Q3C guideline.

### 2.5. In Vitro Cytotoxicity Assay

Human dermal fibroblast (HDF) cells were cultured in Dulbecco’s Modified Eagle’s Medium (DMEM) supplemented with 10% fetal bovine serum (FBS) and 1% penicillin–streptomycin. Cells were maintained at 37 °C in a humidified atmosphere containing 5% CO_2_. For cytotoxicity assessment, HDF cells were seeded into 48-well plates at a density of 1 × 10^4^ cells per well and allowed to adhere for 24 h. After incubation, the culture supernatants were collected for subsequent collagen quantification. Then, 200 µL of fresh medium and 20 µL of EZ-Cytox reagent (EZ-BULK150, DoGenBio, Seoul, Republic of Korea) were added to each well. After 4 h of incubation, cell viability was assessed by measuring absorbance at 450 nm using a microplate reader (INNO-M, LTEK, Seongnam-si, Republic of Korea).

### 2.6. In Vitro Antioxidant Activity (ORAC Assay)

The ORAC assay was performed to evaluate the antioxidant potential of the samples under oxidative stress conditions. Idebenone was prepared at a concentration of 1 mM in 50% acetone, while PCL-based formulations were dissolved in 75 mM phosphate buffer (pH 7.4).

The reaction mixture consisted of 75 mM phosphate buffer containing 80 nM sodium fluorescein. For each assay, 125 µL of the fluorescein solution was combined with 25 µL of the test sample, followed by the addition of 50 µL of 75 mM AAPH (2,2′-azobis(2-methylpropionamidine) dihydrochloride).

After gentle mixing, the reaction plate was pre-incubated at 37 °C for 3 min. Fluorescence intensity was measured every minute for a total duration of 60 min using a microplate reader (excitation: 485 nm; emission: 528 nm). Antioxidant activity was quantified by calculating the area under the fluorescence decay curve (AUC) according to the following formula:

$$AUC=1+\frac{f_{1}}{f_{0}}+\frac{f_{2}}{f_{0}}+\ldots +\frac{f_{60}}{f_{0}}$$

where *f*_0_ represents the fluorescence intensity at the initial time point, and *f*_t_ represents the intensity at each subsequent time point *t*. The net antioxidant capacity was calculated by subtracting the AUC of the control from that of the sample:

$$Net\;AUC={AUC}_{sample}-{AUC}_{control}$$

### 2.7. In Vivo Animal Study

An in vivo study was conducted using SKH-1/60 hairless mice to evaluate the effects of the PEG-PCL-PEG formulation on collagen regeneration. Seven-week-old female mice were obtained from Orient Bio (Seongnam-si, Republic of Korea) and housed under standard laboratory conditions with free access to food and water. All animal procedures were approved by the Institutional Animal Care and Use Committee (IACUC) of CHA Medical University (Approval No. IACUC-230131) and conducted in accordance with the Animal Protection Act and the Guidelines for the Care and Use of Laboratory Animals.

Each mouse received a single dorsal subcutaneous injection of 250 µL of a 10% (*w*/*v*) aqueous PEG-PCL-PEG formulation. Tissue samples were collected at designated time points (1 day, 1 week, 4 weeks, 8 weeks, 12 weeks, 16 weeks, and 24 weeks post-injection) for subsequent gene expression analysis and an evaluation of angiogenic activity. For each time point, eight mice were selected for tissue harvesting and analysis.

### 2.8. Gene Expression Analysis

Quantitative real-time PCR (qRT-PCR) was performed to evaluate the relative mRNA expression levels of target genes using the AccuPower^®^ GreenStar™ qPCR premix (Bioneer, Daejeon, Republic of Korea) and the Exicycler™ real-time PCR system (Bioneer, Daejeon, Republic of Korea). The thermal cycling protocol consisted of an initial denaturation at 96 °C for 5 min, followed by 40 cycles of denaturation at 96 °C for 30 s, annealing at 60 °C for 30 s, and extension at 72 °C for 30 s. Relative gene expression was quantified using the comparative threshold cycle (ΔΔCT) method, with normalization to the housekeeping gene.

### 2.9. Histological Processing and Quantification of Dermal Thickness

Skin tissue samples were fixed in 4% paraformaldehyde (Sigma-Aldrich, St. Louis, MO, USA) at 4 °C and rinsed with PBS for 30 min. Following fixation, samples were cleared in xylene, dehydrated through a graded ethanol series (70%, 80%, and 90%), and embedded in paraffin. Sections were cut at a thickness of 5 µm using a microtome (Thermo Fisher Scientific, Rockford, IL, USA), air-dried, and heat-treated overnight at 65 °C to enhance tissue adhesion.

Histological staining was performed using the hematoxylin and eosin (H&E) and Masson’s trichrome methods. Images were acquired using a digital slide scanner (Axio Scan.Z1, Carl Zeiss, Oberkochen, Germany). Dermal thickness was measured using ImageJ software (version 1.54g; NIH, Bethesda, MD, USA) by selecting four random regions per tissue section. The final dermal thickness for each sample was calculated as the mean of these measurements.

### 2.10. Statistical Analysis

All data are presented as the mean ± standard deviation (SD), based on at least three independent measurements. Statistical significance was evaluated using a one-way analysis of variance (ANOVA), followed by Tukey’s honestly significant difference (HSD) post hoc test for multiple comparisons. A *p*-value of <0.05 was considered statistically significant. All analyses and visualizations were performed using GraphPad Prism (GraphPad Software, San Diego, CA, USA).

## 3. Results and Discussion

### 3.1. Characterization of PEG-PCL-PEG Copolymers

A ring-opening polymerization (ROP) strategy was employed to synthesize amphiphilic PEG-PCL-PEG copolymers with tunable structural features by adjusting the initial molar ratio of CL to PEG. Unlike conventional ROP methods that utilize organometallic catalysts such as stannous octoate (Sn(Oct)_2_), which often require a high temperature and raise concerns regarding residual toxicity, this study adopted a metal-free synthesis approach using HCl as a catalytic monomer activator [30]. This method eliminates contamination from metallic catalysts and meets the high purity and biocompatibility standards required for biomedical polymer synthesis [31].

As a Brønsted acid, HCl efficiently initiates polymerization at room temperature, without the need for an inert atmosphere or specialized solvents. This mild, solvent-efficient, and metal-free system yielded high-purity copolymers with minimal post-processing while offering enhanced control over molecular weight distribution. Such attributes are particularly advantageous for in vivo applications, as they reduce the potential for adverse biological responses associated with catalyst-derived impurities.

**Figure 2:**
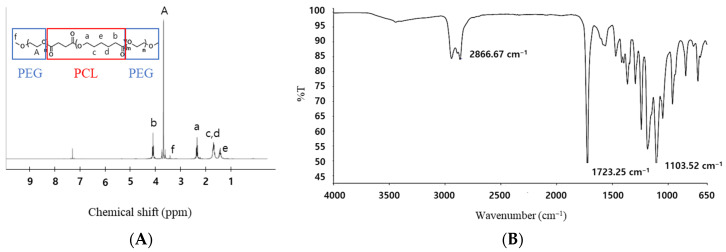
(**A**) ^1^H-NMR spectrum of synthesized PEG-PCL-PEG copolymer in CDCl_3_ (400 MHz), (**B**) FT-IR spectrum showing characteristic stretching vibrations of CH_2_ (2866 cm^−1^), C=O (1723 cm^−1^), and C-O-C (1103 cm^−1^) functional groups.

The structure of the resulting copolymers was characterized using ^1^H-NMR spectroscopy (Figure 2A). Distinct proton signals were observed for both the PEG and PCL segments: a singlet at 3.64 ppm (peak A) corresponding to the –CH_2_CH_2_O– units of PEG and multiple peaks between 4.05 and 1.40 ppm (peaks b, c, d, and e) arising from the methylene protons of the PCL block. The methylene group adjacent to the ester carbonyl (C=O) in PCL appeared at ~2.3 ppm (peak a), and the methoxy terminal group of PEG was observed at ~3.38 ppm (peak f), confirming the successful formation of the tri-block architecture [32]. A minor peak at 7.26 ppm was attributed to residual deuterated chloroform (CDCl_3_), not related to the polymer.

Complementary structural verification was provided by FT-IR spectroscopy (Figure 2B). The FT-IR spectra exhibited characteristic absorption bands for both polymer segments: two distinct peaks were observed near 2866 cm^−1^, corresponding to the symmetric (~2850 cm^−1^) and asymmetric (~2920 cm^−1^) stretching vibrations of –CH_2_– groups, confirming the presence of aliphatic chains in both the PEG and PCL blocks; a sharp peak at 1723 cm^−1^ attributed to ester carbonyl groups in the PCL block; and a notable absorption at 1103 cm^−1^, confirming the C–O–C stretching of PEG ether linkages. These findings are in strong agreement with the ^1^H-NMR results and collectively confirm the successful synthesis of PEG-PCL-PEG tri-block copolymers via the HCl-catalyzed ROP route.

### 3.2. Composition and Molecular Weight Analysis of PEG-PCL-PEG Copolymers

The relative molar composition of the PEG and PCL segments in the synthesized copolymers was quantified by integrating the signal areas in the ^1^H-NMR spectra (Table 1). The experimental block ratios closely matched the theoretical feed ratios, with deviations typically within 2–3%, indicating the efficient incorporation of CL monomers via ring-opening polymerization. This high degree of accuracy confirms that the block composition can be precisely modulated by adjusting the initial monomer feed ratio.

Further insights into the molecular characteristics of the copolymers were obtained using GPC (Figures S1–S8). The number-average molecular weight (M_n_), weight-average molecular weight (M_w_), and polydispersity index (PDI) for each sample are summarized in Table 1. A clear trend was observed wherein lower-molecular-weight samples exhibited minimal divergence between the M_n_ and M_w_ values, while higher-molecular-weight samples showed increased separation between the two values. This divergence reflects the growing influence of high-mass species within the molecular weight distribution, a typical feature of ring-opening polymerization processes.

Despite this variation, all samples maintained PDI values below 1.4, indicating a narrow molecular weight distribution and uniform chain propagation. Notably, the molecular weight values determined by GPC were consistent with the compositional ratios derived from ^1^H-NMR, further supporting that both the chain length and block composition of the copolymers could be reliably controlled through feed molar ratio adjustment.

### 3.3. Aqueous Stability of PEG-PCL-PEG Copolymers

**Figure 3:**
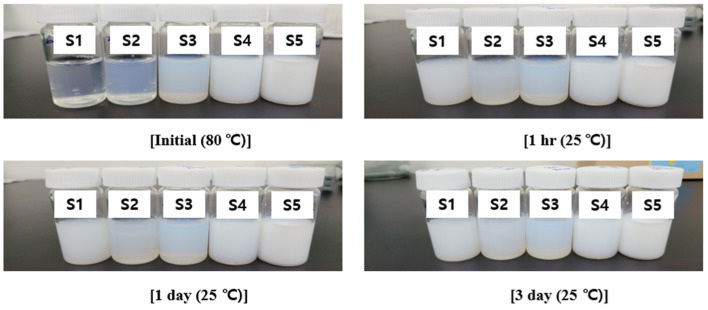
Time-dependent visual turbidity profiles of PEG-PCL-PEG copolymers’ aqueous dispersions (S1–S5) at a concentration of 10% (*w*/*v*). Samples were fully solubilized by heating at 80 °C (top left), and changes in turbidity were monitored during storage at 25 °C for 1 h (top right), 1 day (bottom left), and 3 days (bottom right).

The dispersion stability of PEG-PCL-PEG copolymer samples (S1–S5) in distilled water (10% (*w*/*v*)) was evaluated by monitoring visual turbidity from initial dissolution at 80 °C to storage at 25 °C for three days (Figure 3). While all samples were subjected to heating for solubilization, notable differences in turbidity behavior emerged upon cooling and subsequent storage.

Samples S1 and S2 appeared visually clear at 80 °C but developed noticeable turbidity within 1 day of storage at 25 °C, indicating limited aqueous dispersion stability and a tendency toward phase separation. S4 and S5 showed immediate turbidity even at 80 °C, suggesting incomplete solubilization due to excessive hydrophobic PCL content. These samples exhibited visible sedimentation over time, reflecting poor water compatibility and thermal stability. In contrast, S3 maintained consistent turbidity with no apparent phase separation or precipitation over the entire test period. The well-balanced ratio of hydrophilic PEG and hydrophobic PCL segments in S3 contributes to the formation of a stable colloidal dispersion. Based on these observations, S3 was selected as the optimal formulation for the further synthesis and biological evaluation of PEG-PCL-PEG tri-block copolymers.

### 3.4. Thermal Properties and the Effects of Precipitation Temperature

**Figure 4:**
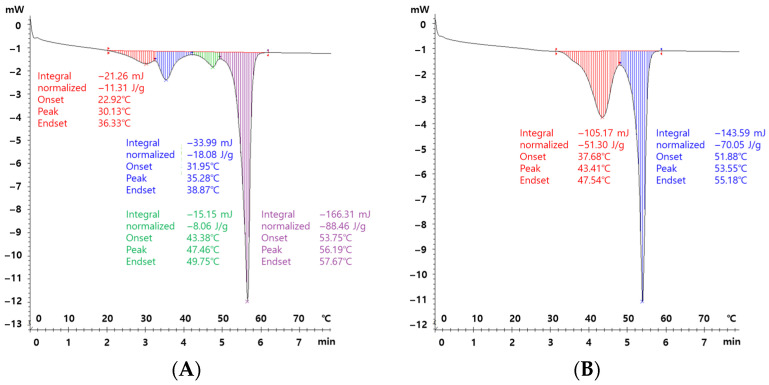
DSC thermograms of PEG-PCL-PEG copolymers formed under different precipitation temperatures: (**A**) 15 °C and (**B**) 5 °C. All copolymers were synthesized using identical polymerization and solvent conditions, with only the precipitation temperature being varied. This comparison aims to evaluate the effects of temperature on thermal transitions, including the crystallinity and melting behavior of the copolymers.

The thermal behavior of PEG-PCL-PEG copolymers precipitated at different temperatures was examined by DSC, as shown in Figure 4. Precipitation temperature played a critical role in determining both the crystallization characteristics and the purity of the resulting materials. In the sample precipitated at 15 °C, four endothermic transitions were observed, appearing at approximately 30 °C, 35 °C, 45 °C, and 55 °C. The peaks near 45 °C and 55 °C correspond to the melting temperatures of the PEG and PCL segments, respectively, in agreement with the previously reported thermal properties of these polymers [33]. In contrast, the additional lower-temperature transitions (~30 °C and ~35 °C) are not characteristic of PEG or PCL crystallinity and are more likely attributed to unreacted monomers or low-molecular-weight byproducts that co-precipitated under the slower phase separation dynamics at the elevated temperature [34]. By contrast, the thermogram of the copolymer precipitated at 5 °C displayed only two sharp peaks corresponding to the PEG and PCL melting transitions, with no additional thermal events. This simplified thermal profile suggests that rapid phase separation at lower temperature effectively minimized the incorporation of residual impurities and unreacted species into the polymer matrix. These impurities are presumed to have remained in the supernatant rather than becoming entrapped during polymer solidification.

Overall, these results indicate that lower-temperature precipitation not only promotes the formation of well-defined crystalline domains but also enhances the purity of the final product by minimizing the co-precipitation of low-molecular-weight species. Precipitation at 5 °C thus appears to optimize both the thermal uniformity and structural integrity of the synthesized PEG-PCL-PEG copolymers.

### 3.5. Residual Solvent Analysis

GC was employed to evaluate the residual content of organic solvents in the final PEG-PCL-PEG copolymers, ensuring compliance with safety and regulatory standards. The targeted solvents, diethyl ether, methylene chloride, and toluene, were selected based on their usage during the synthesis and purification steps and assessed according to the ICH Q3C guidelines (Table 2). All detected concentrations were substantially lower than the respective permissible thresholds, with diethyl ether measured at 22 ppm (limit: ≤5000 ppm), methylene chloride at 55 ppm (limit: ≤600 ppm), and toluene at 223 ppm (limit: ≤890 ppm).

These results indicate that the employed purification and drying protocols were highly effective in minimizing solvent residues. Notably, the low residual levels may also be linked to the optimized low-temperature precipitation strategy adopted during the synthesis and purification steps. As previously evidenced by DSC analysis, samples precipitated at 5 °C showed a reduced presence of low-molecular-weight impurities, suggesting that rapid phase separation at lower temperatures curtailed the entrapment of unreacted species and solvent remnants. Collectively, the GC and DSC results underscore the critical role of precipitation temperature in enhancing both the structural integrity and chemical purity of the copolymers. This supports their potential suitability for biomedical and cosmetic applications, where stringent quality and safety standards must be met.

### 3.6. Visual Characteristics of PEG-PCL-PEG-Based Formulations

**Figure 5:**
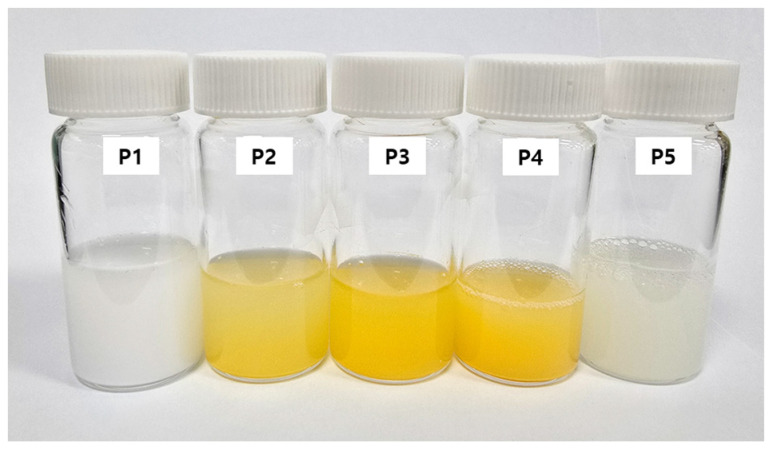
Photographic comparison of PEG-PCL-PEG-based formulations (P1–P5) showing visual changes due to addition of idebenone, HA, and glycerin.

The visual appearances of the prepared formulations are shown in Figure 5, while their corresponding compositional details are provided in Table 3. Each sample was dispersed in distilled water according to its respective composition. Formulation P1, composed solely of PEG-PCL-PEG without additional additives, exhibited a milky and opaque appearance, reflecting the intrinsic turbidity of the polymer matrix. In contrast, formulations P2, P3, and P4, each containing idebenone, displayed a distinct yellow hue, corresponding to the inherent coloration of the antioxidant compound. Slight variations in color intensity and opacity were noted among these samples, influenced by the concentration of idebenone and the addition of sodium hyaluronate in P4. Formulation P5, representing the regeneration-oriented series, showed a semi-translucent, whitish appearance. Compared to P1, its reduced turbidity is likely due to the inclusion of sodium hyaluronate and glycerin, which modulate the refractive index and enhance dispersion uniformity [35,36,37]. These visual observations provide preliminary evidence of the successful additive incorporation and clearly distinguish the antioxidant and regeneration-focused formulations. This differentiation supports the strategic formulation design intended for subsequent biological evaluation.

### 3.7. In Vitro Cytocompatibility of Idebenone Formulations

**Figure 6:**
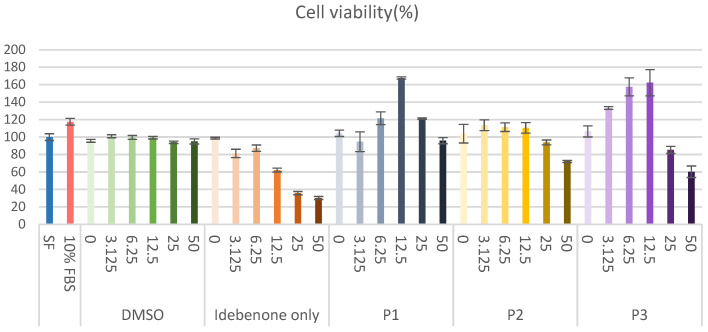
Cell viability of HaCaT cells after treatment with varying concentrations of free idebenone and PEG-PCL-PEG-based formulations (P1–P3).

Prior to evaluating antioxidant efficacy, a cell viability assay was conducted to assess the cytocompatibility of idebenone-containing formulations. HaCaT cells were exposed to serial dilutions of free idebenone or PEG–PCL–PEG-based formulations (P1–P3), and cell viability was measured after 24 h using a WST-based assay (Figure 6). Free idebenone induced a dose-dependent reduction in viability, with significant cytotoxicity observed at concentrations above 2.5 µg/mL, where cell viability dropped below 50%. This finding highlights the potential toxicity risks associated with free idebenone at higher doses. In contrast, formulations P1–P3 exhibited markedly improved cytocompatibility across all tested concentrations. At the highest tested concentration (50 µg/mL), P2 and P3 maintained cell viability at approximately 70% and 60%, respectively, whereas free idebenone caused a marked decline in cell viability under the same conditions. This protective effect is likely due to the encapsulation of idebenone within the PEG-PCL-PEG copolymer matrix, which may regulate its release profile and reduce direct cellular exposure [38,39,40,41]. Overall, these findings support the use of PEG-PCL-PEG-based formulations, as they enable safer and more biocompatible delivery of idebenone, providing a promising platform for subsequent functional assessments.

### 3.8. Antioxidant Performance of Idebenone-Loaded Formulations

**Figure 7:**
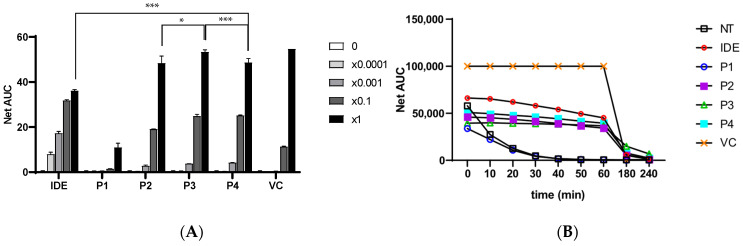
Antioxidant activity and retention of idebenone-loaded PEG-PCL-PEG formulations evaluation by ORAC assay. (**A**) Net AUC values reflecting dose-dependent antioxidant effects of formulations P2-P4 and controls (IDE: free idebenone; VC: vitamin C). Statistical significance at ×1 concentration was determined using one-way ANOVA followed by Tukey’s HSD post hoc test (* *p* < 0.05, *** *p* < 0.001). (**B**) Antioxidant activity retention over 240 min (NT: no treatment control). P3 demonstrated most sustained activity, while P4 retained acceptable efficacy with added regenerative benefits from HA.

The antioxidant efficacy of idebenone-loaded PEG–PCL–PEG formulations was evaluated using the ORAC assay, with the results summarized in Figure 7. Both the initial radical scavenging capacity and the time-dependent retention of antioxidant activity were assessed through net area under the curve (Net AUC) analysis. As shown in Figure 7A, idebenone-containing formulations (P2–P4) exhibited clear concentration-dependent increases in antioxidant activity, whereas the polymer-only control (P1) showed minimal effects. A slight elevation in Net AUC was observed for P1 at the lowest concentration, likely due to optical interference from sample turbidity rather than true antioxidant action. Among all formulations, P3 consistently achieved the highest Net AUC values across most concentrations, indicating superior free radical scavenging activity attributed to its higher idebenone content (0.1% (*w*/*v*)).

Interestingly, formulation P4, which contained the same idebenone concentration as P3, with the addition of 0.1% (*w*/*v*) sodium hyaluronate (HA) resulted in a modest reduction in Net AUC. This attenuation of peak antioxidant activity may be attributed to physicochemical interactions between HA and idebenone. First, HA increases the formulation’s overall viscosity, which could slow molecular diffusion and limit idebenone’s accessibility to reactive oxygen species (ROS) [42,43,44,45]. Second, the hydrophilic polymer network of HA may sterically hinder the release of idebenone from the micellar core or interact through hydrogen bonding, reducing the effective availability of free antioxidant molecules [46,47]. These mechanisms suggest that while HA enhances hydration and formulation stability, it can modulate the release kinetics and functional activity of lipophilic compounds such as idebenone.

### 3.9. Gene Expression in Dermal Remodeling

**Figure 8:**
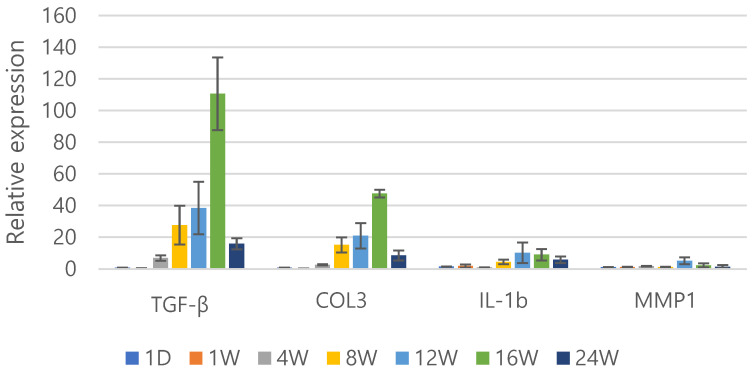
Comparative gene expression analysis of TGF-β, COL3, IL-1β, and MMP1 over 24 weeks following PEG-PCL-PEG formulation injection.

To investigate the regenerative efficacy of the PEG–PCL–PEG formulation, the gene expression profiles of the key genes involved in extracellular matrix remodeling were assessed over a 24-week period post-injection (Figure 8). The analysis focused on TGF-β, COL3, interleukin-1β (IL-1β), and matrix metalloproteinase-1 (MMP1), which are closely linked to collagen synthesis, tissue remodeling, and inflammatory responses. TGF-β expression exhibited a significant increase, peaking at 16 weeks with a notably higher level than the initial baseline. This marked elevation suggests the robust stimulation of fibroblast activation and matrix production during mid-phase remodeling. COL3 expression also increased over time, showing a delayed but coordinated rise that culminated around the same time point as that for TGF-β. The synchronous upregulation of these two markers implies active collagen biosynthesis and sustained tissue regeneration.

In contrast, IL-1β and MMP1, markers associated with inflammation and matrix degradation, showed a steady decline following an initial transient response. IL-1β expression decreased notably after the first week and remained suppressed throughout the observation period, suggesting the establishment of a low-inflammatory microenvironment. A similar pattern was observed with MMP1, indicating the attenuation of matrix breakdown activity and the enhanced structural stability of the regenerated tissue. Collectively, the gene expression profiles highlight the dual regenerative potential of the PEG-PCL-PEG formulation: it activates pro-regenerative signaling early on while concurrently downregulating catabolic and inflammatory mediators. This temporal regulation supports its role in orchestrating a favorable environment for long-term dermal remodeling.

### 3.10. Macroscopic Tissue Remodeling and Angiogenesis Post-Injection

**Figure 9:**
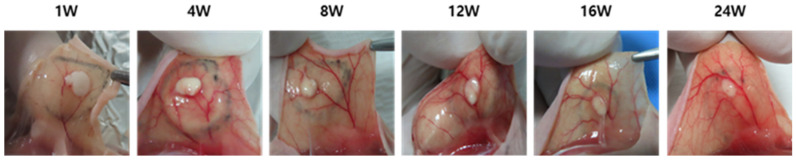
Time-dependent morphological changes and angiogenesis following subcutaneous injection of PEG-PCL-PEG formulation in hairless mice over 24 weeks.

Building upon the gene expression findings, sequential morphological changes and angiogenesis at the injection site were evaluated over a 24-week period (Figure 9). The early upregulation of regenerative markers such as TGF-β and COL3, as identified in the molecular analyses, indicated the activation of extracellular matrix synthesis and tissue remodeling pathways [48,49,50]. These signals correlated well with the macroscopic and histological changes observed during this study.

At 1-week post-injection, the implanted PEG-PCL-PEG formulation remained largely intact, with minimal evidence of vascularization, corresponding to the initial phase of TGF-β pathway activation. By 4 weeks, initial signs of angiogenesis emerged, concurrent with rising COL3 expression, indicative of nascent extracellular matrix formation and host tissue integration. At 8 weeks, vascular density had significantly increased, aligning with the peak expression levels of TGF-β and COL3, suggesting robust neovascularization and active remodeling. Evaluations at 12 and 16 weeks revealed a further maturation of the vascular network, along with the progressive resorption of the material, paralleling the sustained downregulation of inflammatory mediators such as IL-1β and MMP1. By 24 weeks, the injection site displayed a dense, well-organized capillary network with minimal residual material, indicating the completion of tissue remodeling and stabilization. Overall, these morphological findings corroborate the molecular data, demonstrating that the PEG-PCL-PEG formulation effectively promotes a regenerative microenvironment that supports both sustained angiogenesis and dermal tissue regeneration.

### 3.11. Histological Assessment of Collagen Regeneration and Dermal Remodeling

**Figure 10:**
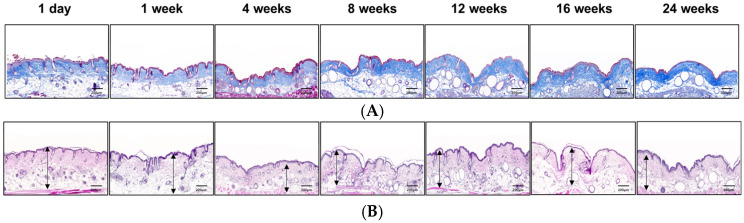
Histological evaluation of collagen regeneration and dermal remodeling following subcutaneous injection of PEG-PCL-PEG copolymers. (**A**) Masson’s trichrome staining demonstrates progressive collagen deposition (blue) and extracellular matrix remodeling over 24 weeks. (**B**) H&E staining reveals sustained dermal thickness (black arrows) and tissue integration with reduced inflammation.

The histological progression of collagen regeneration following the subcutaneous injection of the PEG-PCL-PEG formulation was evaluated using Masson’s trichrome and hematoxylin and eosin (H&E) staining at multiple time points, as shown in Figure 10. In the Masson’s trichrome-stained sections (Figure 10A), collagen fibers appeared blue, allowing for a qualitative assessment of ECM deposition. At 1 day and 1 week post-injection, collagen density was low, characterized by loosely arranged and discontinuous fibers within the dermis. By 4 and 8 weeks, a noticeable increase in collagen fiber density and organization was observed, correlating with the peak expression of regenerative markers such as TGF-β and COL3. At 12 weeks, the dermal matrix exhibited substantial enhancement, with densely packed, well-organized collagen bundles indicative of robust matrix remodeling. This mature collagen architecture persisted through 16 and 24 weeks, suggesting the establishment of a stabilized and long-lasting ECM network.

Complementary findings were evident in H&E-stained sections (Figure 10B). In the early stages (1 day and 1 week), the dermis appeared relatively thin, populated by sparsely distributed fibroblasts. A transient reduction in dermal thickness was observed at 4 weeks, reflecting the tissue remodeling phase associated with matrix resorption and new ECM synthesis. By 8 and 12 weeks, dermal thickening became prominent, accompanied by increased cellularity and matrix compaction, reflecting active fibroblast proliferation and extracellular matrix production. This mature dermal architecture was maintained through 16 and 24 weeks without signs of regression. Together, the histological results strongly aligned with the molecular profiles and dermal thickness, confirming that the PEG-PCL-PEG formulation promotes sustained collagen regeneration, active matrix remodeling, and long-term dermal stabilization. The concordance among gene expression patterns, dermal thickness evolution, and histological architecture underscores the regenerative efficacy and translational promise of this injectable skin booster platform.

## 4. Conclusions

This study reports the development of an injectable PEG-PCL-PEG-based platform that integrates both antioxidant and regenerative functionalities within a biocompatible polymer matrix. The synthesized copolymers exhibited high structural fidelity and chemical purity, supporting their suitability for biomedical use. Idebenone-loaded formulations showed effective antioxidant activity while maintaining favorable cytocompatibility, particularly when encapsulated in the polymer matrix. The inclusion of HA slightly reduced peak antioxidant activity but contributed to extended activity and regenerative potential. The HA-containing formulation promoted tissue remodeling in vivo, as demonstrated by gene expression, histological analysis, and dermal thickening trends. Collectively, the results highlight the PEG-PCL-PEG injectable system as a versatile and promising candidate for advanced skin therapeutics, offering a tunable foundation for applications in cosmetic dermatology and regenerative medicine.

## 5. Patent

Application no. 10-2023-0048231 (Republic of Korea).

## Figures and Tables

**Table 1:** Feed composition, molar ratios, and molecular weight of PEG-PCL-PEG tri-block copolymers synthesized with varying PEG and ε-caprolactone (CL) ratios.

Sample Code	Molecular Weight (PEG-PCL)	Molar Ratio of CL/PEG	Feed Molar Ratio of CL-PEG	GPC (M_w_/M_n_/PDI)
S1	2000-1000	8.8	9	4612/3924/1.18
S2	2000-2000	18.4	18.2	6222/4555/1.36
S3	2000-4000	35.7	36	8004/6077/1.32
S4	2000-6000	54.1	53	10,360/7289/1.42
S5	2000-10,000	88.6	88	12,188/8237/1.48
S6	5000-7500	74.3	66	11,547/8587/1.34
S7	5000-10,000	89.4	88	12,857/9455/1.36
S8	2000-8000-2000	77.2	-	11,175/8684/1.29

**Table 2:** Residual solvent content in synthesized PEG-PCL-PEG copolymers as determined by GC.

Residual Solvent	ICH Class	Criteria (ppm)	Result (ppm)
Diethyl ether	Class 3	≤5000	22
Methylene chloride	Class 2	≤600	55
Toluene	Class 2	≤890	223

**Table 3:** Composition of PEG-PCL-PEG-based injectable formulations incorporating antioxidant and regenerative components.

Composition	P1	P2	P3	P4	P5
PEG-PCL-PEG (2k-8k-2k) (% (*w*/*v*))	10	10	10	10	20
Idebenone (% (*w*/*v*))	-	0.05	0.1	0.1	-
Sodium hyaluronate (1.4) (% (*w*/*v*))	-	-	-	0.1	1
Glycerin (% (*w*/*v*))	-	-	-	-	1
Arginine (% (*w*/*v*))	-	-	-	-	0.1
DI water (mL)	100	100	100	100	100

## Data Availability

The data presented in this study are available upon request from the corresponding author.

## Supplementary Materials

The following supporting information can be downloaded at: https://www.mdpi.com/article/10.3390/polym17141892/s1, Figure S1: GPC chromatogram of sample S1; Figure S2: GPC chromatogram of sample S2; Figure S3: GPC chromatogram of sample S3; Figure S4: GPC chromatogram of sample S4; Figure S5: GPC chromatogram of sample S5; Figure S6: GPC chromatogram of sample S6; Figure S7: GPC chromatogram of sample S7; Figure S8: GPC chromatogram of sample S8.

## References

[1] Trovato F., Ceccarelli S., Michelini S., Vespasiani G., Guida S., Galadari H.I., Nisticò S.P., Colonna L., Pellacani G. (2024). Advancements in regenerative medicine for aesthetic dermatology: A comprehensive review and future trends. Cosmetics.

[2] Zhao Y., Zhang X., Liu W., Wang S., Wu J. (2023). Global Market Trends of Non-Invasive Aesthetic Procedures and Their Biomedical Implications. Aesthet. Plast. Surg..

[3] Christen M.-O., Vercesi F. (2020). Polycaprolactone: How a well-known and futuristic polymer has become an innovative collagen-stimulator in esthetics. Clin. Cosmet. Investig. Dermatol..

[4] Mochane M.J., Mokhena T.C., Sadiku E.R. (2019). Nature-derived and synthetic additives to poly(ɛ-caprolactone) nanofibers: A review. Front. Chem..

[5] Homaeigohar S., Boccaccini A.R. (2022). Nature-derived and synthetic additives to poly(ɛ-caprolactone) nanofibrous systems for biomedicine: An updated overview. Front. Chem..

[6] Yaseri R., Fadaie M., Mirzaei E., Samadian H., Ebrahiminezhad A. (2023). Surface modification of polycaprolactone nanofibers through hydrolysis and aminolysis: A comparative study on structural characteristics, mechanical properties, and cellular performance. Sci. Rep..

[7] Kayan G.Ö., Kayan A. (2023). Polycaprolactone composites/blends and their applications especially in water treatment. Chem. Eng..

[8] utikov A.B., Song J. (2015). Biodegradable PEG-based amphiphilic block copolymers for tissue engineering applications. ACS Biomater. Sci. Eng..

[9] Perin F., Motta A., Maniglio D. (2021). Amphiphilic copolymers in biomedical applications: Synthesis routes and property control. Mater. Sci. Eng. C.

[10] Cardona Y.V., Muñoz L.G., Cardozo D.G., Chamorro A.F. (2024). Recent applications of amphiphilic copolymers in drug release systems for skin treatment. Pharmaceutics.

[11] Kuperkar K., Patel D., Atanase L.I., Bahadur P. (2022). Amphiphilic block copolymers: Their structures, and self-assembly to polymeric micelles and polymersomes as drug delivery vehicles. Polymers.

[12] Papakonstantinou E., Roth M., Karakiulakis G. (2012). Hyaluronic acid: A key molecule in skin aging. Derm.-Endocrinol..

[13] Dovedytis M., Liu Z.J., Bartlett S. (2020). Hyaluronic acid and its biomedical applications: A review. Eng. Regen..

[14] Wang S.T., Neo B.H., Betts R.J. (2021). Glycosaminoglycans: Sweet as sugar targets for topical skin anti-aging. Clin. Cosmet. Investig. Dermatol..

[15] Sundaram H., Mackiewicz N., Burton E., Peno-Mazzarino L., Lati E., Meunier S. (2016). Pilot comparative study of the topical action of a novel, crosslinked resilient hyaluronic acid on skin hydration and barrier function in a dynamic, three-dimensional human explant model. J. Drugs Dermatol..

[16] Milani M., Sparavigna A. (2017). The 24-hour skin hydration and barrier function effects of a hyaluronic 1%, glycerin 5%, and *Centella asiatica* stem cells extract moisturizing fluid: An intra-subject, randomized, assessor-blinded study. Clin. Cosmet. Investig. Dermatol..

[17] Wittmann F., Prix N., Mayr S., Angele P., Wichmann M.W., van den Engel N.K., Hernandez-Richter T., Chaudry I.H., Jauch K.W., Angele M.K. (2005). L-arginine improves wound healing after trauma-hemorrhage by increasing collagen synthesis. J. Trauma Acute Care Surg..

[18] Jerônimo M.S., Barros A.P., Morita V.E.Z., Alves E.O., de Souza N.L.B., de Almeida R.M., Nóbrega Y.K.M., Cavalcanti Neto F.F., Amorin R., Borin M.F. (2016). Oral or topical administration of L-arginine changes the expression of TGF and iNOS and results in early wounds healing. Acta Cir. Bras..

[19] Witte M.B., Barbul A. (2003). Arginine physiology and its implication for wound healing. Wound Repair Regen..

[20] Suárez-Rivero J.M., Pastor-Maldonado C.J., Povea-Cabello S., Álvarez-Córdoba M., Villalón-García I., Munuera-Cabeza M., Suárez-Carrillo A., Talaverón-Rey M., Sánchez-Alcázar J.A. (2021). Coenzyme Q10 analogues: Benefits and challenges for therapeutics. Antioxidants.

[21] Gueven N., Ravishankar P., Eri R., Rybalka E. (2021). Idebenone: When an Antioxidant Is Not an Antioxidant. Redox Biol..

[22] Tibbitt M.W., Dahlman J.E., Langer R. (2016). Emerging Frontiers in Drug Delivery. J. Am. Chem. Soc..

[23] Makadia H.K., Siegel S.J. (2011). Poly Lactic-co-Glycolic Acid (PLGA) as Biodegradable Controlled Drug Delivery Carrier. Polymers.

[24] Burdick J.A., Prestwich G.D. (2011). Hyaluronic Acid Hydrogels for Biomedical Applications. Adv. Mater..

[25] Ghorai S.K., Maji S., Subramanian B., Maiti T.K., Chattopadhyay S. (2019). Promoted osteoconduction of polyurethane–urea based 3D nanohybrid scaffold through nanohydroxyapatite adorned hierarchical titanium phosphate. ACS Appl. Bio Mater..

[26] Ma Y., Hu N., Liu J., Zhai X., Wu M., Hu C., Li L., Lai Y., Pan H., Lu W.W. (2019). Three-dimensional printing of biodegradable piperazine-based polyurethane-urea scaffolds with enhanced osteogenesis for bone regeneration. ACS Appl. Mater. Interfaces.

[27] Jeong B., Bae Y.H., Kim S.W. (1997). Thermoreversible Gelation of PEG–PLGA–PEG Triblock Copolymer Aqueous Solutions. Macromol. Rapid Commun..

[28] Wang H., Tong Z., Li Y., Liu G., Liu X., Liu F., Zhang W. (2013). In Vivo Biocompatibility and Degradation Behavior of PEG-PCL-PEG Hydrogel as an Injectable Carrier for Controlled Drug Delivery. Polym. Degrad. Stab..

[29] Lee S.H., Shin H., Jo S., Cho I.-S., Kim H.-W. (2018). PEG–PCL–PEG Hydrogel-Embedded Fibroblasts for Enhanced Dermal Collagen Synthesis and Skin Tissue Engineering. J. Biomed. Mater. Res. A.

[30] Chueasupcharoen W., Meepowpan P., Manokruang K., Sriyai M. (2024). Metal-Free Ring-Opening Polymerization for the Synthesis of Biocompatible Star-Shaped Block Copolymers with Controllable Architecture. Eur. Polym. J..

[31] Xu J., Song J., Pispas S., Zhang G. (2014). Metal-Free Controlled Ring-Opening Polymerization of ε-Caprolactone in Bulk Using Tris(pentafluorophenyl)borane as Catalyst. Polym. Chem..

[32] Huang Y., Li L., Li G. (2015). An Enzyme-Catalyzed Access to Amphiphilic Triblock Copolymer of PCL-b-PEG-b-PCL: Synthesis, Characterization and Self-Assembly Properties. Polym. Chem..

[33] Jeong B., Bae Y.H., Lee D.S., Kim S.W. (1997). Biodegradable Block Copolymers as Injectable Drug-Delivery Systems. Nature.

[34] Liu Y., Tong Z., Wang J., Zhao Y., Zhang D., Li Y., Qiao M., Chen D. (2019). One-Pot Synthesis and Characterization of Biodegradable PEG-PCL Copolymers with Narrow PDI for Drug Delivery. Nanomaterials.

[35] Kurt A.A., Aslan İ. (2025). A Novel Liposomal In-Situ Hydrogel Formulation of *Hypericum perforatum* L.: In Vitro Characterization and In Vivo Wound Healing Studies. Gels.

[36] Smułek W., Grząbka-Zasadzińska A., Kilian A., Szczepaniak O., Król E. (2023). Design of Vitamin-Loaded Emulsions in Agar Hydrogel Matrix Dispersed with Plant Surfactants. Food Biosci..

[37] Daryab M., Faizi M., Mahboubi A., Daryab M., Ghaffari F., Rezaei F. (2022). Preparation and Characterization of Lidocaine-Loaded, Microemulsion-Based Topical Gels. Iran. J. Pharm. Res..

[38] Odrobińska J., Skonieczna M., Neugebauer D. (2020). PEG Graft Polymer Carriers of Antioxidants: In Vitro Evaluation for Transdermal Delivery. Pharmaceutics.

[39] Yang H.S., Yu S., Kim J., Baek K.J., Lee Y.R., Lee H.S. (2022). Facile Solvent-Free Preparation of Antioxidant Idebenone-Loaded Nanoparticles for Efficient Wound Healing. Pharmaceutics.

[40] Patole M.S., Pokharkar V.B. (2015). Cellular Interactions and Photoprotective Effects of Idebenone-Loaded Nanostructured Lipid Carriers Stabilized Using PEG-Free Surfactant. Int. J. Pharm..

[41] Alves P.L.M., Nieri V., Moreli F.C., Constantino E., de Souza J., Oshima-Franco Y., Grotto D. (2024). Unveiling New Horizons: Advancing Technologies in Cosmeceuticals for Anti-Aging Solutions. Molecules.

[42] Gaynanova G., Vasileva L., Kashapov R., Kuznetsova D., Kushnazarova R., Tyryshkina A., Vasilieva E., Petrov K., Zakharova L., Sinyashin O. (2021). Self-Assembling Drug Formulations with Tunable Permeability and Biodegradability. Molecules.

[43] Witika B.A., Poka M.S., Demana P.H., Matafwali S.K., Metlamane S., Khalmanga S.M.M., Makoni P.A. (2022). Lipid-Based Nanocarriers for Neurological Disorders: A Review. Pharmaceutics.

[44] Chiriac A.P., Rusu A.G., Nita L.E., Chiriac V.M., Neamtu I. (2021). Polymeric Carriers Designed for Encapsulation of Essential Oils with Biological Activity. Pharmaceutics.

[45] Montenegro L., Turnaturi R., Parenti C., Pasquinucci L. (2018). Idebenone: Novel Strategies to Improve Its Systemic and Local Efficacy. Nanomaterials.

[46] Mayol L., Biondi M., Russo L., Malle B.M., Schwach-Abdetllaoui K., Borzacchiello A. (2014). Amphiphilic Hyaluronic Acid Derivatives Toward the Design of Micelles for the Sustained Delivery of Hydrophobic Drugs. Carbohydr. Polym..

[47] Berillo D., Zharkinbekov Z., Kim Y., Raziyeva K., Temirkhanova K., Saparov A. (2021). Stimuli-Responsive Polymers for Transdermal, Transmucosal and Ocular Drug Delivery. Pharmaceutics.

[48] Guillamat-Prats R. (2021). The Role of MSC in Wound Healing, Scarring and Regeneration. Cells.

[49] Rong N., Mistriotis P., Wang X., Tseropoulos G., Rajabian N., Zhang Y., Wang J., Liu S., Andreadis S.T. (2019). Restoring Extracellular Matrix Synthesis in Senescent Stem Cells. FASEB J..

[50] Tan P.-C., Zhou S.-B., Ou M.-Y., He J.-Z., Zhang P.-Q., Zhang X.-J., Xie Y., Gao Y.-M., Zhang T.-Y., Li Q.F. (2022). Mechanical Stretching Can Modify the Papillary Dermis Pattern and Papillary Fibroblast Characteristics During Skin Regeneration. J. Investig. Dermatol..

