# Supplementary material for: Injectable PEG-PCL-PEG Copolymers for Skin Rejuvenation: In Vitro Cell Studies to in Vivo Collagen Induction
Source: Polymers (Basel). 2025 Jul 8;17(14):1892. doi: 10.3390/polym17141892 (PMC12300825; doi:10.3390/polym17141892)
Supplement: Supplementary file 1 [file polymers-17-01892-s001.zip › polymers-3674832-supplementary.pdf]

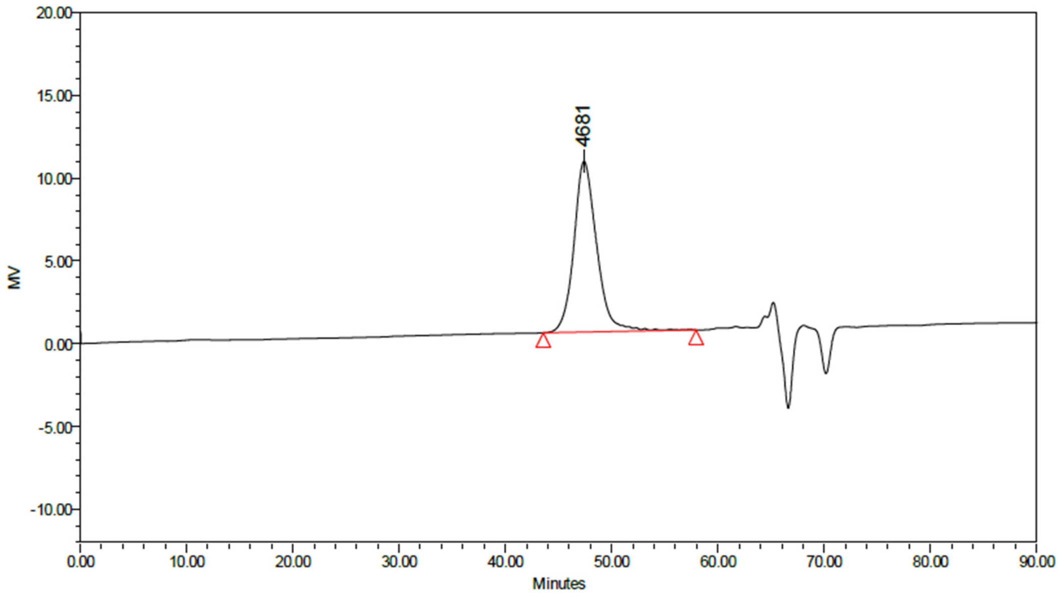

GPC Results

	Mn	Mw	MP (Daltons)	Polydispersity	% Area
1	3924	4612	4681	1.175508	100.0

Figure S1. GPC chromatogram of sample S1

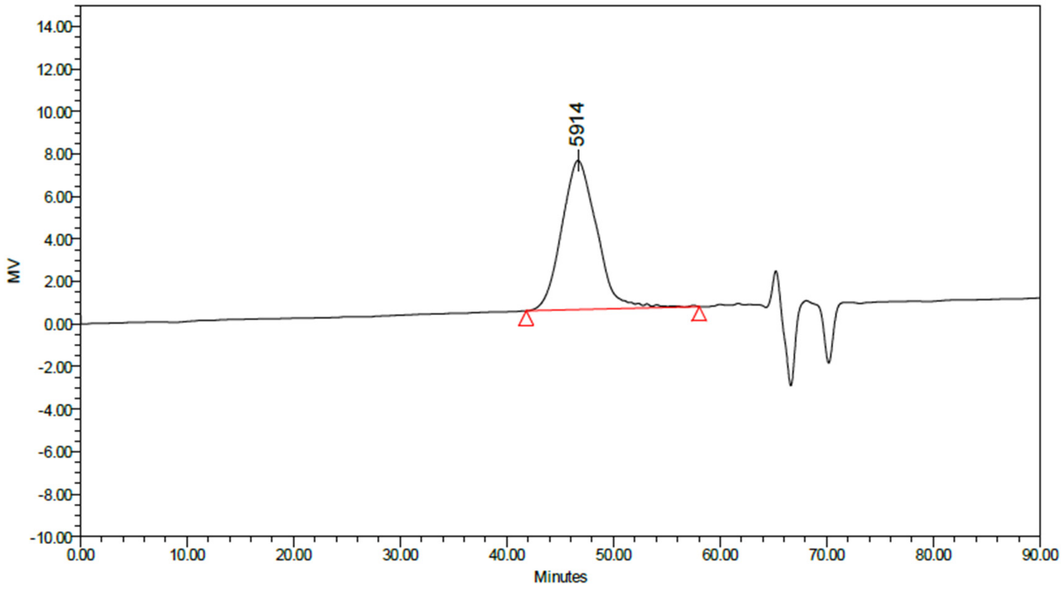

GPC Results

	Mn	Mw	MP (Daltons)	Polydispersity	% Area
1	4555	6222	5914	1.366033	100.0

Figure S2. GPC chromatogram of sample S2

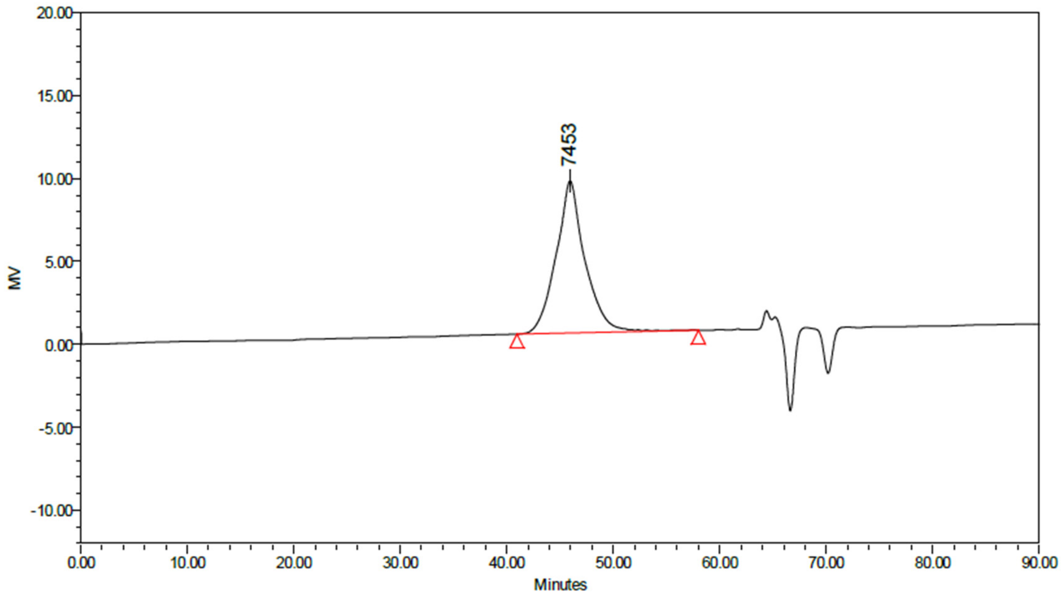

GPC Results

	Mn	Mw	MP (Daltons)	Polydispersity	% Area
1	6077	8004	7453	1.316981	100.0

Figure S3. GPC chromatogram of sample S3

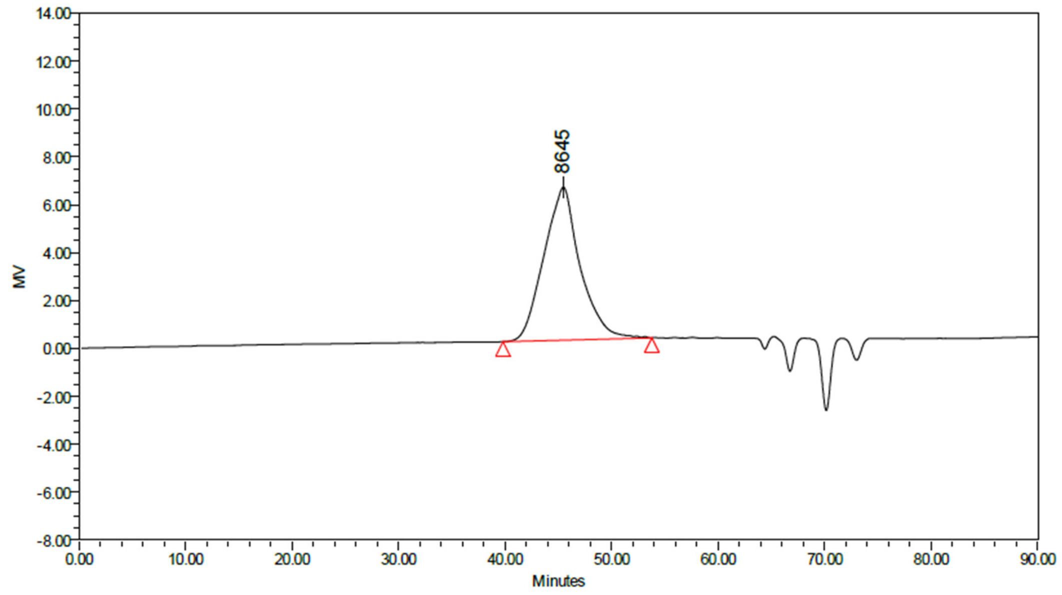

GPC Results

	Mn	Mw	MP (Daltons)	Polydispersity	% Area
1	7289	10360	8645	1.421287	100.0

Figure S4. GPC chromatogram of sample S4

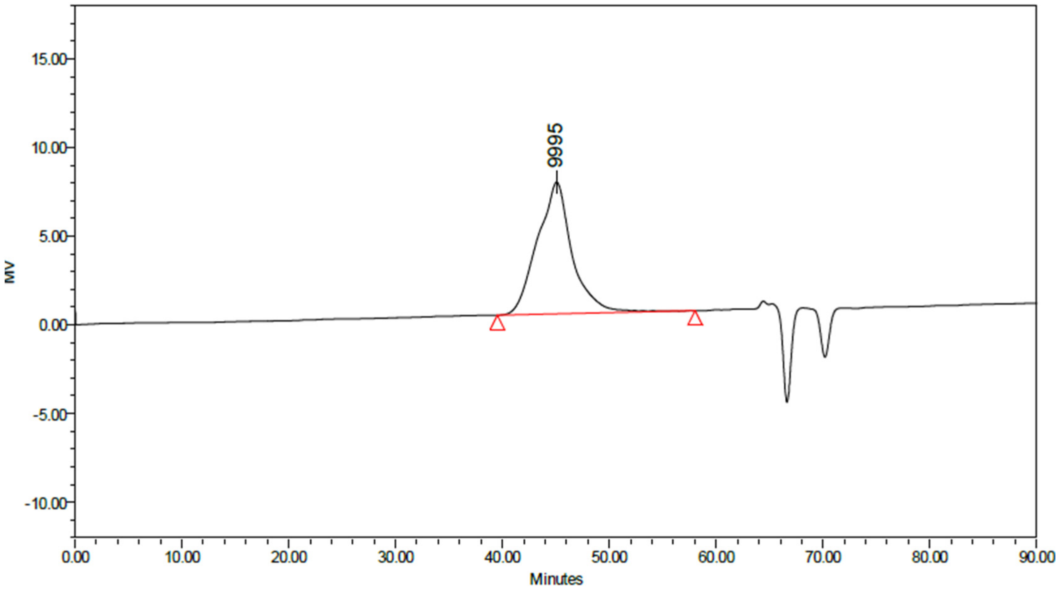

GPC Results

	Mn	Mw	MP (Daltons)	Polydispersity	% Area
1	8237	12188	9995	1.479614	100.0

Figure S5. GPC chromatogram of sample S5

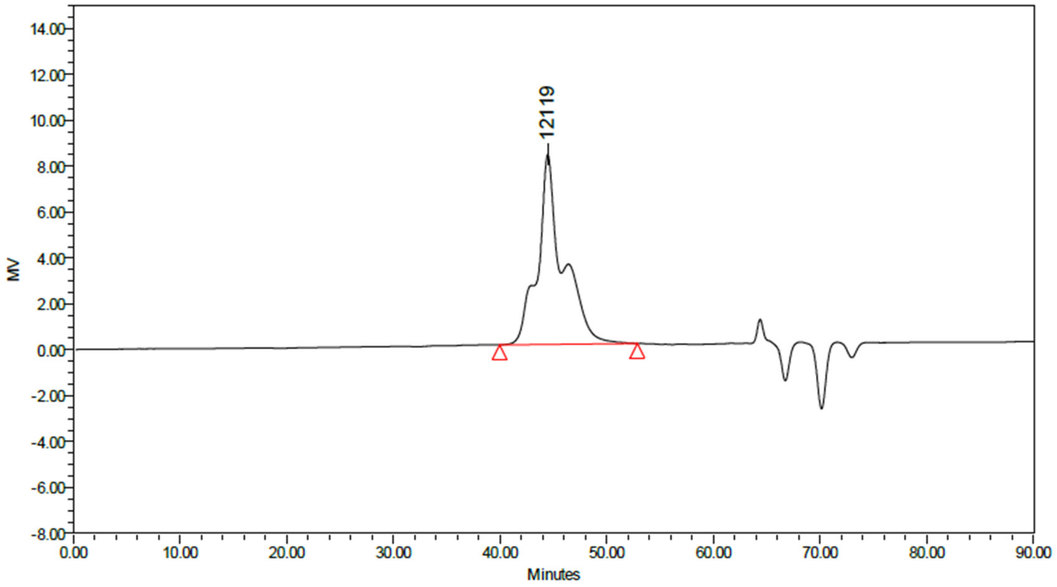

GPC Results

	Mn	Mw	MP (Daltons)	Polydispersity	% Area
1	8587	11547	12119	1.344808	100.0

Figure S6. GPC chromatogram of sample S6

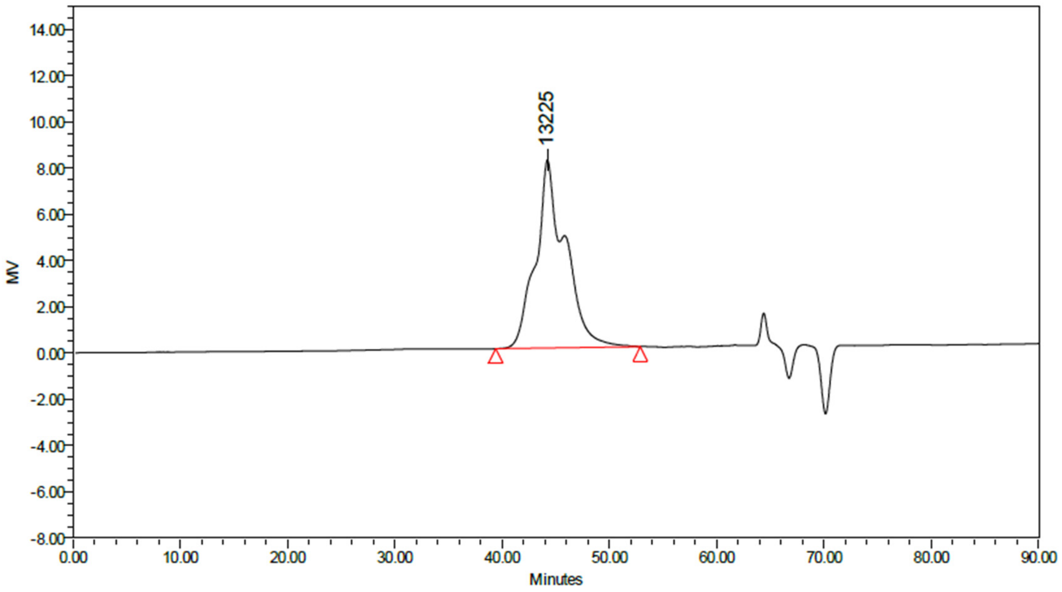

GPC Results

	Mn	Mw	MP (Daltons)	Polydispersity	% Area
1	9455	12857	13225	1.359700	100.0

Figure S7. GPC chromatogram of sample S7

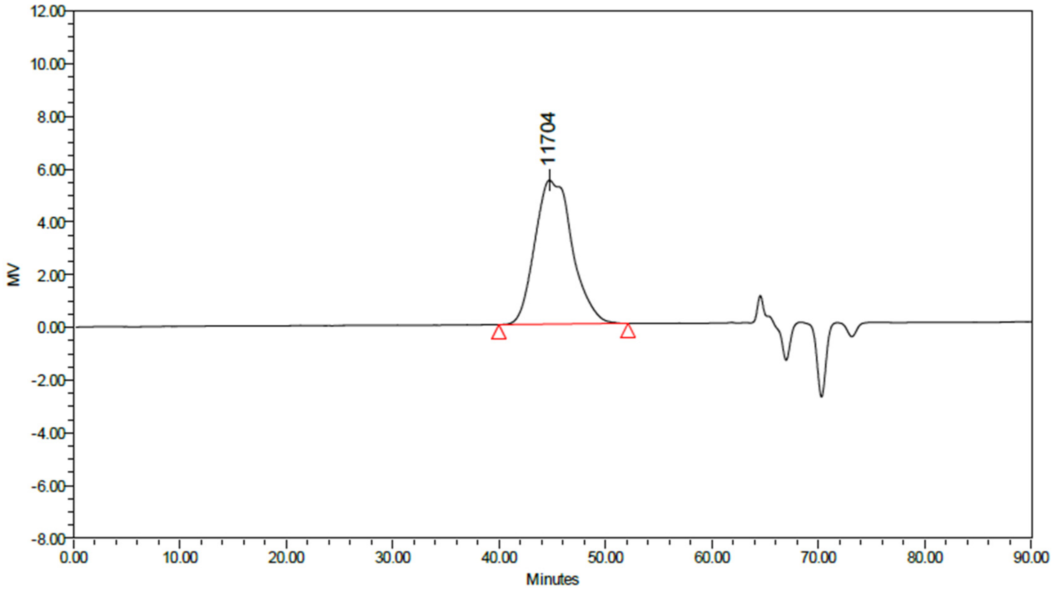

GPC Results

	Mn	Mw	MP (Daltons)	Polydispersity	% Area
1	8684	11175	11704	1.286832	100.0

Figure S8. GPC chromatogram of sample S8
